# Printed Flexible Thermoelectric Nanocomposites Based on Carbon Nanotubes and Polyaniline

**DOI:** 10.3390/ma14154122

**Published:** 2021-07-24

**Authors:** Marcin Słoma, Maciej Andrzej Głód, Bartłomiej Wałpuski

**Affiliations:** Institute of Metrology and Biomedical Engineering, Warsaw University of Technology, 8 sw. A. Boboli St., 02-525 Warsaw, Poland; glod.maciek@gmail.com (M.A.G.); b.walpuski@mchtr.pw.edu.pl (B.W.)

**Keywords:** thermoelectric generators, nanotubes, polyaniline, flexible electronics, Seebeck coefficient

## Abstract

A new era of composite organic materials, nanomaterials, and printed electronics is emerging to the applications of thermoelectric generators (TEGs). Special attention is focused on carbon nanomaterials and conducting polymers, and the possibility to form pastes and inks for various low-cost deposition techniques. In this work, we present a novel approach to the processing of composite materials for screen-printing based on carbon nanotubes (CNTs) and polyaniline (PANI), supported with a dielectric polymer vehicle. Three different types of such tailor-made materials were prepared, with a functional phase consisted of carbon nanotubes and polyaniline composites fabricated with two methods: dry mixing of PANI CNT powders and in situ polymerisation of PANI with CNT. These materials were printed on flexible polymer substrates, exhibiting outstanding mechanical properties. The best parameters obtained for elaborated materials were $\sigma =405.45\;S\cdot m^{-1}$, $S=15.4\;\mu V\cdot K^{-1}$, and $PF=85.2\;\mathrm{nW}\cdot m^{-1}K^{-2}$, respectively.

## 1. Introduction

The demand for renewable energy in the current era of climate change is an important issue for scientists and industry. One of the ways to solve this problem is efficient energy distribution and consumption without excessive energy waste. A thermoelectric generator (TEG) is a device that enables recovery of the energy from lost heat and converts it directly to electrical energy with the use of special thermoelectric materials, usually in the form of solid-state metal and oxide semiconductor compounds [1]. Currently, most of the energy produced by humans is irretrievably lost as heat, escaping into the atmosphere [2]. TEG generators can partially prevent this process. They have several advantages such as lack of moving parts and noiseless operation [3]. There are many low-temperature heat sources where thermoelectric generators are used such as automotive, industrial, and human body heat [4,5]. TEG is based on the Seebeck phenomenon involving the diffusion of high-energy electrons located at a higher temperature towards a cooler zone of the material [6]. Important parameters describing thermoelectric structures include Seebeck coefficient S (ΔV/ΔT), determining the amount of generated voltage for a given temperature difference; electrical conductivity σ ($S\cdot m^{-1}$), determining the material’s ability to conduct electricity; power factor PF ($W\cdot m^{-1}K^{-2}$), determining the amount of power generated at a given temperature difference; the figure of merit ZT, describing the relation between materials electrical and thermal conductivity, all of which allow the comparison of device and material efficiency. Conventional inorganic thermoelectric materials exhibit good performance, but on the other hand, usually have poor mechanical properties [7], making their industrial applications limited. The development of organic thermoelectric materials could allow the elaboration of many new industrial applications due to their easy processing with other materials into composites.

TEG generators can be fabricated with various technologies such as thin-film technology [8], thick-film technology [9], or semiconductor technology [10]. For the fabrication of organic TEGs, thick-film technologies such as screen printing [11,12,13] and spray coating [14] are most commonly used. Although the thermoelectric properties of conductive polymers are usually poor, they can be easily improved by combining them with other materials such as carbon nanotubes (CNTs) [8,15,16]. Carbon nanotubes, due to their electrical and mechanical properties, are excellent as a functional phase of thermoelectric nanocomposites. Composites based on carbon nanomaterials and conducting polymers feature a much simpler fabrication process than conventional semiconducting inorganic thermoelectric materials. The fabrication of composites is most suitable for thick-film technology. They require fewer resources than thin-film technologies or the fabrication of inorganic solid TEGs (such as a high vacuum). To produce a composite for thick-film technology, ink or paste consisting of a functional phase and a polymer matrix must be prepared. Hong et al. fabricated organic thermoelectric films consisting of CNTs and P3HT, using spray coating. With 50 wt.% of CNTs, the following values of Seebeck coefficient, electrical conductivity, and PF at room temperature were obtained: $102\pm 3\;\mu V\cdot K^{-1}$, $224\pm 19\;S\cdot {\mathrm{cm}}^{-1}$, and $231\pm 19\;\mu W\cdot m^{-1}K^{-2\;}$[14]. Wei et al. fabricated polymer thermoelectric modules with screen printing, from PEDOT:PSS paste. At the temperature of 25 °C, $S=18\;\mu V\cdot K^{-1}$ and $PF=25\;\mu W\cdot m^{-1}K^{-2}$ were obtained, while at 200 °C, the thermoelectric parameters were $S=25{\;\mu \mathrm{VK}}^{-1}$ and $PF=34\;\mu W\cdot m^{-1}K^{-2}$, respectively [13]. Zhang et al. fabricated a thermoelectric generator with a roll-to-roll technique in which the n-type thermolegs were composed of graphene, while the p-type ones were composed of PEDOT:PSS. Such generators at a temperature difference of ΔT = 10 °C generated power of $0.24\;\mathrm{mW}\cdot m^{-2}$ [9].

Extensive research is currently conducted on the use of carbon nanomaterials for TEGs. Hewitt et al. conducted a study on the influence of single-walled carbon nanotubes (SWCNTs) content in a composite with PVDF matrix. The electrical conductivity and Seebeck coefficient of films containing 5 wt.% up to 100 wt.% of SWCNTs were investigated. The best electrical conductivity was obtained for samples containing 100 wt.% of SWCNTs $\sigma >10^{4}\;S\cdot m^{-1}$, while the highest Seebeck coefficient was obtained for 5 wt.%, $S=32\;\mu V\cdot K^{-1}$, respectively. The high SWCNT content in the composite material influences the thermal conductivity value (the more nanotubes the higher conductivity), leading to a decrease in the Seebeck coefficient [17]. Zhang et al. conducted an extensive literature review presenting the current findings on organic thermoelectric materials. One-dimensional materials exhibit better thermoelectric properties, compared to multidimensional materials [18]. To obtain better thermoelectric performance, it is necessary to combine CNTs with other materials due to the high thermal conductivity of CNTs. The most common composites are based on conductive polymers. They reduce the thermal conductivity of the composite material, having a small negative effect on the electrical conductivity compared to dielectric polymers. Polyaniline (PANI) exhibits some of the best properties among organic materials combined with CNTs [19]. Jeong et al. performed an extensive analysis of the existing development concerning thermoelectric composites and generators based on these materials. Similarly, the combination of CNTs and PANI was found to be one of the most promising in terms of TEG generators. Among the cited works, the highest power factor for the PANI/CNT composite was $PF=217\;\mu W\cdot m^{-1}K^{-2}$ [20], while the highest Seebeck coefficient was $S=65\;\mu V\cdot K^{-1}$ [21], respectively. SWCNT monolayer exhibits good electrical properties, presenting several potential fabrication methods to prepare TEG generators, also with thick-film technology [18]. Wang et al. prepared a summary of achievements in the field of organic TEGs. They characterised the types of nanotubes, along with their fabrication methods such as chemical vapor deposition, high-pressure carbon monoxide method, or arc discharge method, in terms of electrical conductivity and Seebeck coefficient. Methods for fabrication of finished TEG modules were also presented such as large-area continuously synthesised CNT films and localised doping technology. In addition, the effect of CNT diameter on thermoelectric properties was presented, and the smaller the diameter of the CNT was, the higher the Seebeck coefficient was [15].

## 2. Materials and Methods

The aim of this work is to compare the thermoelectric properties of composites with functional phases containing CNT/PANI made by in situ polymerisation and by mixing PANI and CNT powders. The in situ polymerisation process is a common method described in the literature used for the fabrication of CNT/PANI composites [22].

Two solutions were prepared during the chemical polymerisation of aniline. The first one contained 125 mL of 1.5 M HCl from Stanlab (Lublin, Poland) and 12.5 g of ammonium persulfate (APS) from Chempur (Piekary Śląskie, Poland) with a molar mass 228.3 g/mol, while the second one consisted of 125 mL of 1.5 M HCl and 10 mL of aniline purchased from Sigma-Aldrich (Poznań, Poland). To mix the substances efficiently, both liquids were placed in an ultrasonic bath. The aniline solution was additionally in an ice bath to maintain a low temperature below 5 °C. The liquid containing APS was added gradually with a speed of 2–3 drops per second. During the mixing, the colour changes of the liquid were observed as follows: light brown, dark blue, ink colour, dark green. The mixed solutions were stirred for an additional 4 h at low temperature. In the next step, the substance was filtered with 1.5 M HCl and distilled water to obtain a colourless material and was left for 24 h at room temperature, and later dried in an oven at 50 °C for 12 h. After drying, a dark green powder of emeraldine salt was obtained.

In the next step, in situ chemical polymerisation of aniline together with CNTs was performed. For this purpose, two solutions were prepared. The first consisted of 125 mL of 1.5 M HCl, 10 mL of aniline, and 6.81 g of industrial-grade multiwalled CNTs from Nanocyl (Sambreville, Belgium). The second contained 125 mL of 1.5 M HCl and 12.5 g of APS. The solution with CNTs was placed in an ice bath to maintain a low temperature and was subjected to continuous stirring. The second solution was gradually added (2–3 drops per second) with continuous stirring. The liquid was then stirred for another 4 h after which the suspension containing CNT and PANI was filtered with 1.5 M HCl and distilled water until a colourless filtrate was obtained. The substance was left for 24 h at room temperature after which it was dried at 50 °C for 12 h. Thus, a powder containing 40 wt.% of CNTs and 60 wt.% of PANI was obtained.

A common way to obtain composites with new properties is mixing the two different powders or a solution-based approach of adding functional filler to polymer solution in a solvent. Kale et al. used a polyurethane matrix mixed with graphene oxide and silica nanofillers to fabricate with screen printing conductive paths with improved mechanical properties [23]. Cho et al. prepared a composite consisting of ${\mathrm{RuO}}_{2}/\mathrm{PEDOT}:\mathrm{PSS}/\mathrm{graphene}$, also produced by screen printing which led to the enhancement of electrical performance [24]. Here, we present a new approach to preparing thermoelectric composite materials consisted of three components. In addition to functional CNT and PANI, very often described in the literature for fabrication of organic thermolegs in TEGs, poly(methyl methacrylate) (PMMA) was added in our approach to improve the mechanical properties and adhesion to the flexible substrate.

The dried polyaniline was mixed with CNTs in a ratio of 40 wt.% CNTs and 60 wt.% PANI. Then, a paste was prepared with the CNT/PANI composite blend as the functional phase and PMMA polymer in 2-(2-butoxyethoxy)ethyl acetate (OKB) organic solvent as a polymer vehicle. This unusual approach for TEGs is common for the fabrication of screen-printed pastes, and here, the goal was to produce a paste with appropriate rheology for this technique. The paste consisted of 2.5 g of PANI, 1.66 g of CNTs, and 63.79 g of polymer vehicle, of which 6 wt.% was PMMA and 94 wt.% OKB. Considering that all the solvent evaporates during the drying process, a composite containing 3.83 g of polymer matrix and 4.16 g of functional phase was obtained. In the final composite material, the functional filler constituted 52.1 wt.% and the polymer matrix 47.9 wt.%. The product of this composition is denoted as $S1_{\mathrm{mix}}$. A 25 mm × 2 mm rectangular template was used for printing. Samples were dried in the oven at 50 °C for 12 h. The thickness of the samples was measured using a Taylor Hobson Form Talysurf PGI 830 profilometer (Leicester, UK). The thickness of printed samples was estimated to be 50 ± 10 μm. Silver contacts were deposited at the ends of the composite strips using fast-drying conductive silver ink.

The other approach was to prepare a composite paste for screen printing with a PMMA polymer vehicle and in situ polymerised PANI-CNT composite. The same as previously the goal was to produce a paste with appropriate rheology for this technique. While the viscosity of such samples was lower than for previously prepared pastes, we decided to add a more functional phase. The final composition contained 4 g of CNT/PANI functional phase and 15.946 g of a solution containing 6 wt.% PMMA in 94 wt.% OKB; after solvent evaporation, this results in a composite with 81 wt.% of functional phase and 19 wt.% of the polymer matrix. Thickness was determined to be 140 ± 20 μm. The composite samples with the above composition were denoted as $S2_{\mathrm{insitu}}$.

A second sample was also fabricated with a functional phase made by in situ polymerisation but with lower filler content. The amount of polymer vehicle was identical to the previous approach, while the CNT/PANI composite content was reduced to 3.8 g. The weight ratio of the composite was 79.8% of the filler to 20.2% of the polymer matrix. The average thickness of printed samples after drying was determined to be 140 ± 20 μm. The composite samples described here are denoted as $S3_{\mathrm{insitu}}$.

## 3. Results and Discussions

**Figure 1:**
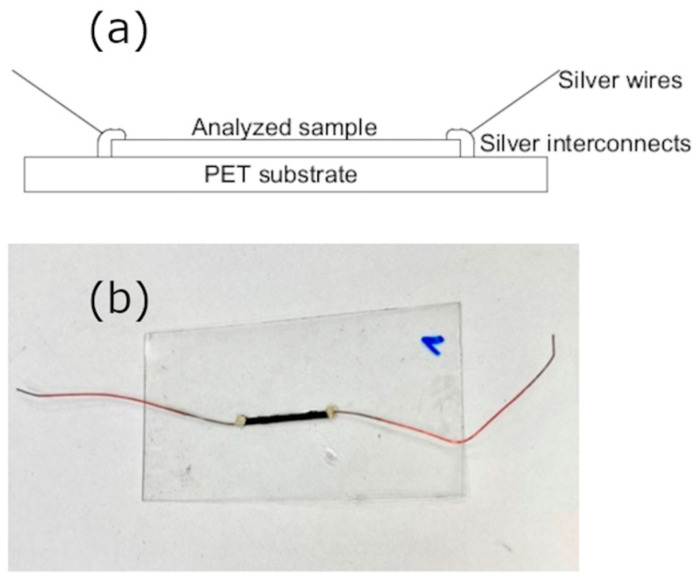
(**a**) is a schematic drawing of the designed thermoelectric structure and (**b**) is the picture of a screen-printed sample for electrical and thermal measurements.

Figure 1a presents the schematic drawing of the designed thermoelectric structure, and Figure 1b the picture of the fabricated structure. It was printed on the flexible PET foil and silver wires (copper plated) were attached with a fast-drying conductive silver paste. The wires were added to facilitate the measurements of the electrical parameters.

The rheology of the pastes was the key parameter to be investigated before printing. Shear rates value was 1501 1/s, which is typical for the screen-printing process [25]. The viscosity values of the materials were measured. For $S2_{\mathrm{insitu}}$ sample containing 81 wt.% of functional phase and $S3_{\mathrm{insitu}}$ sample containing 79.8 wt.%, despite the small difference in filler percentage, the viscosity differed by more than five times. It is expected that for samples based on in situ polymerisation at 75 wt.% of the functional phase, similar rheology to the $S1_{\mathrm{mix}}$ paste would be obtained. The results of the viscosity measurements are presented in Table 1.

Despite the differences in rheological properties between the $S2_{\mathrm{insitu}}$, $S3_{\mathrm{insitu}}$, and $S1_{\mathrm{mix}}$ pastes, no drastic difference was observed during the screen-printing process. The $S2_{\mathrm{insitu}}$ and $S3_{\mathrm{insitu}}$ materials exhibited a greater tendency to form agglomerates, leading to negligible flaws in printed samples. A small adjustment in the printing angle of the squeegee eliminated this disadvantage. For the screen-printing technique, the value of viscosity should be in the range between 2 and 40 Pa·s [25]. For the $S2_{\mathrm{insitu}}$ sample, the viscosity was slightly out of this range; however, it was still possible to print this material with minor adjustments to the printing process.

**Figure 2:**
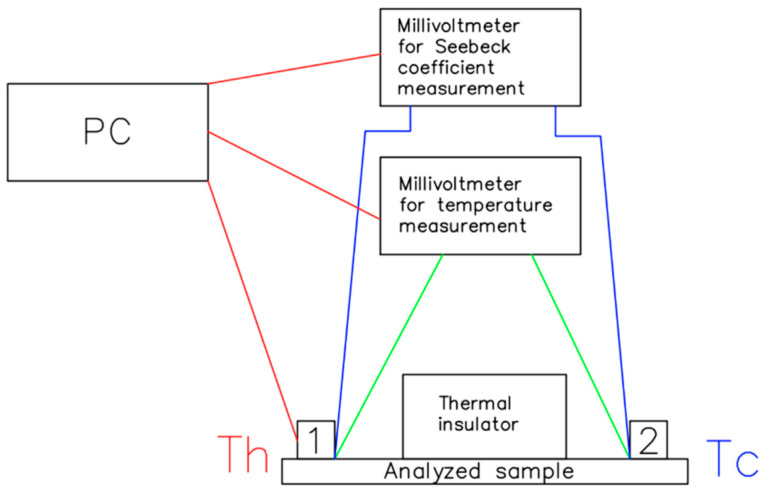
Schematic drawing of the measuring station of the Seebeck coefficient, in which 1 stands for the electric heater, and 2 stands for the ice bath.

After printing and drying, several tests were conducted to determine the electric and thermoelectric properties of the obtained materials. First, the electrical resistance was measured using Keithley 2010 multimetre (Beaverton, OR, USA). At least seven samples of $S1_{\mathrm{mix}}$, $S2_{\mathrm{insitu}}$, and $S3_{\mathrm{insitu}}$ materials were prepared. The final values of parameters are the arithmetic means values of the tested samples. Knowing the resistance R and dimensions of the samples, the electrical conductivity σ was calculated using the following formula:

(1) σ=l/(A·R)

where *A* is the cross-sectional area, and *l* is the length of the sample. Then, an automated measuring laboratory stand was prepared for Seebeck coefficient measurements controlled by the LabVIEW 2015 dedicated software, presented in Figure 2. The temperature difference between the ends of the sample was ΔT=100 K. An electric heater is controlled by a PID regulator, while 0 °C is realised with the ice bath. For temperature measurements, a type k thermocouple attached to the multimetre was used. The second multimetre was used to measure thermoelectric voltage used for the Seebeck coefficient calculations.

Knowing the Seebeck coefficient values *S* and electrical conductivity *σ*, the power factor *PF* could be calculated from the following formula [26]:

(2) $$PF=\sigma \cdot S^{2}\;$$

The obtained results are shown in Table 2.

Considering the fact that industrial-grade multiwalled carbon nanotubes were used during composite fabrication without additional purification of segregation procedures, the obtained results were satisfactory. Wang et al. prepared a PANI/MWCNT composite, which at 40 wt.% of CNTs had a Seebeck coefficient of 10 μV/K [27], which is lower than the value obtained in this work. Despite the presence of a nonconducting PMMA polymer improving mechanical properties, the obtained Seebeck coefficients were of the same order of magnitude for all obtained samples as in many other works on PANI/CNT composites [20,27,28,29,30,31]. Replacing used MWCNTs with higher quality SWCNTs would significantly improve thermoelectric properties, especially in terms of PF [32]. Our main objective was to compare alternative methods of producing the functional phase of the composite and not to obtain the best possible parameters; therefore, it was not necessary to use the highest-grade CNTs.

The functional phase produced by in situ polymerisation was characterised by a higher volume of CNTs, compared to dry mixed CNTs and PANI powders. This most likely indicates the localisation of PANI agglomerates on CNTs, as observed by Wang et al. [27]. For the functional filler produced by the in situ method, a significant increase in viscosity (more than five times) was observed between 79.8 wt.% and 81 wt.% samples $S2_{\mathrm{insitu}}$ and $S3_{\mathrm{insitu}}$ samples. The observed increase in conductivity with higher-loaded paste is due to the more conductive material in the composite, which is in line with the common understanding of conductive composites based on the percolation model. However, this is combined with an increase in thermal conductivity of the composite due to the high content of nanotubes being one of the best thermal conductors. This results in the decrease in the Seebeck coefficient by the increased content of CNTs, which have both good electrical and thermal conductivity.

$S1_{\mathrm{mix}}$ samples had the highest electrical conductivity of the elaborated composites, even though they did not have the lowest electrical resistance. This is due to their dimensions. After drying, $S1_{\mathrm{mix}}$ samples had almost three times lower thickness than $S2_{\mathrm{insitu}}$ and $S3_{\mathrm{insitu}}$, with the same width and length for all three. The lower filler content is combined with a higher PMMA/OKB solution amount and thus more solvent in the paste, which evaporates during drying.

Another important parameter for TEGs is the thermoelectric power factor PF. The highest value was recorded for sample $S1_{\mathrm{mix}}$, and it was more than two times higher than for $S2_{\mathrm{insitu}}$ and almost three times higher than for $S3_{\mathrm{insitu}}$. We observed that as the Seebeck coefficient increases, PF decreases for fabricated samples. This is due to the electrical conductivity of the materials. For all three composites, the Seebeck coefficient was similar (only a 0.9 μV/K difference). Considering that the Seebeck coefficients of all materials were similar, the electrical conductivity plays a key role in determining PF.

**Figure 3:**
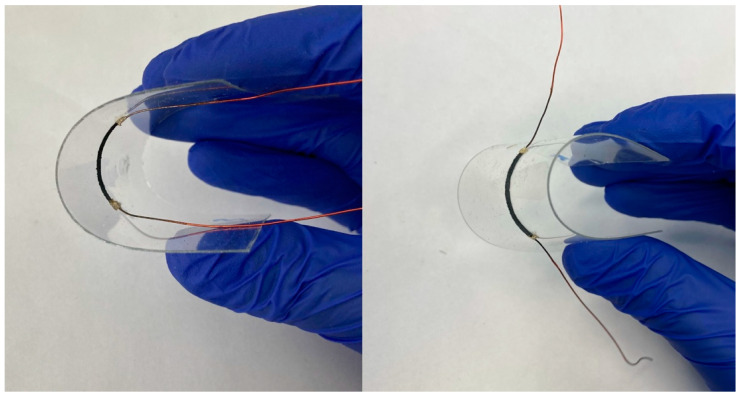
Flexible thermoelectric composites bent to the radius of 5 mm both ways (inner side and outer side of the substrate).

PANI generally has poor mechanical properties [33]. There are many methods to improve the adhesion of PANI to the substrate [34]. However, our alternative approach was focused on the modification of the composition by adding additional polymer. Saadattalab et al. analysed the mechanical properties of PANI/CNT composites. At low CNT content in the composite material, almost twofold improvement in tensile strength was observed [35]. Our novel approach of triple material-based composition allowed us to prepare flexible thermoelectric generators on flexible PET foils, retaining their electrical properties even at the curvature radius of 5 mm, regardless of the bending direction (Figure 3).

## 4. Conclusions

During this work, organic flexible thermoelectric generators (TEGs) were prepared on a PET substrate from elaborated printed composite pastes with carbon nanotubes and conducting polymer. In addition, two methods of fabrication of the functional phase were investigated: one based on the dry mixing of PANI and CNT powders and the second based on the in situ polymerisation of PANI and CNTs together. Elaborated materials based on the mixing of PANI and CNT powders performed better than materials from in situ polymerisation; despite a lower amount of the functional phase, better electrical conductivity and better PF were obtained. When industrial-grade MWCNTs were used, compared with the literature results, the obtained thermoelectric properties were satisfactory and even outperforming high-purity SWCNT-based TEGs. In this study, the best thermoelectric properties were as follows: $\sigma =405.45\;S\cdot m^{-1}$, $S=15.4\;\mu V\cdot K^{-1}$, and $\mathrm{PF}=85.2\;\mathrm{nW}\cdot m^{-1}K^{-2}$. The advantage of the novel approach presented in this work is the improvement of mechanical properties by the presence of PMMA in the composite. At the same time, Seebeck coefficients were of the same order of magnitude as in many other works on PANI/CNT composites.

## Figures and Tables

**Table 1:** Rheological parameters of obtained thermoelectric pastes, measured at the room temperature (21.3 °C).

Sample	Shear Rate (1/s)	Viscosity (Pa·s)
$S1_{mix}$	153.2	2.7
$S2_{insitu}$	155.2	73.6
$S3_{insitu}$	153.2	13.2

**Table 2:** Electric and thermoelectric properties of obtained composites.

	Resistance (Ω)	Electrical Conductivity (S/m)	Seebeck Coefficient (μV/K)	$\mathrm{PF}\;\left(\mathrm{nw}/mK^{2}\right)$
$S1_{mix}$	616.6	405.5	14.5	85.2
$S2_{insitu}$	525.3	170	14.9	37.7
$S3_{insitu}$	734.5	121.6	15.4	28.8

## Data Availability

The data that support the findings of this study are available from the corresponding author upon reasonable request.
